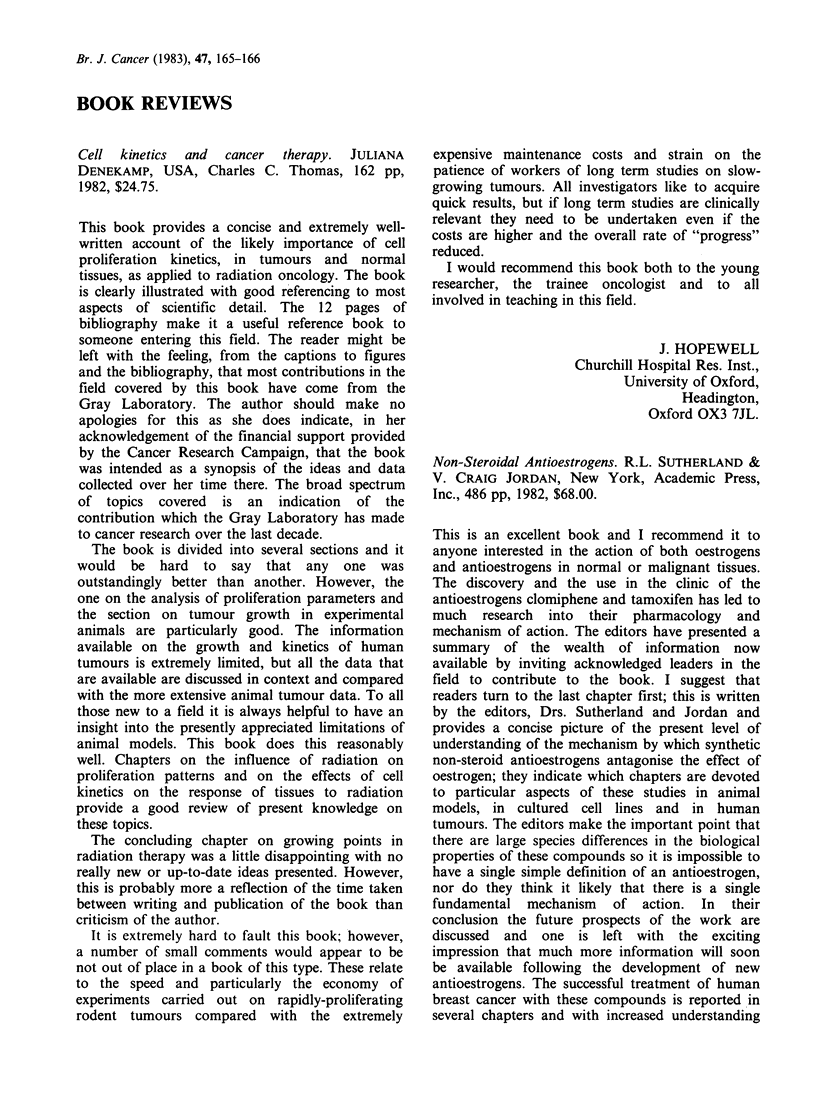# Cell kinetics and cancer therapy

**Published:** 1983-01

**Authors:** J. Hopewell


*Br. J. Cancer* (1983), **47**, 165–166

### BOOK REVIEWS

*Cell kinetics and cancer therapy.* JULIANA DENEKAMP, USA, Charles C. Thomas, 162 pp, 1982, $24.75.

This book provides a concise and extremely well-written account of the likely importance of cell proliferation kinetics, in tumours and normal tissues, as applied to radiation oncology. The book is clearly illustrated with good referencing to most aspects of scientific detail. The 12 pages of bibliography make it a useful reference book to someone entering this field. The reader might be left with the feeling, from the captions to figures and the bibliography, that most contributions in the field covered by this book have come from the Gray Laboratory. The author should make no apologies for this as she does indicate, in her acknowledgement of the financial support provided by the Cancer Research Campaign, that the book was intended as a synopsis of the ideas and data collected over her time there. The broad spectrum of topics covered is an indication of the contribution which the Gray Laboratory has made to cancer research over the last decade.

The book is divided into several sections and it would be hard to say that any one was outstandingly better than another. However, the one on the analysis of proliferation parameters and the section on tumour growth in experimental animals are particularly good. The information available on the growth and kinetics of human tumours is extremely limited, but all the data that are available are discussed in context and compared with the more extensive animal tumour data. To all those new to a field it is always helpful to have an insight into the presently appreciated limitations of animal models. This book does this reasonably well. Chapters on the influence of radiation on proliferation patterns and on the effects of cell kinetics on the response of tissues to radiation provide a good review of present knowledge on these topics.

The concluding chapter on growing points in radiation therapy was a little disappointing with no really new or up-to-date ideas presented. However, this is probably more a reflection of the time taken between writing and publication of the book than criticism of the author.

It is extremely hard to fault this book; however, a number of small comments would appear to be not out of place in a book of this type. These relate to the speed and particularly the economy of experiments carried out on rapidly-proliferating rodent tumours compared with the extremely expensive maintenance costs and strain on the patience of workers of long term studies on slow-growing tumours. All investigators like to acquire quick results, but if long term studies are clinically relevant they need to be undertaken even if the costs are higher and the overall rate of "progress" reduced.

I would recommend this book both to the young researcher, the trainee oncologist and to all involved in teaching in this field.

J. HOPEWELL
Churchill Hospital Res. Inst.,
University of Oxford,
Headington,
Oxford OX3 7JL.